# The Dopamine Assisted Synthesis of MoO_3_/Carbon Electrodes With Enhanced Capacitance in Aqueous Electrolyte

**DOI:** 10.3389/fchem.2022.873462

**Published:** 2022-04-19

**Authors:** Nazgol Norouzi, Darrell Omo-Lamai, Farbod Alimohammadi, Timofey Averianov, Jason Kuang, Shan Yan, Lei Wang, Eli Stavitski, Denis Leshchev, Kenneth J. Takeuchi, Esther S. Takeuchi, Amy C. Marschilok, David C. Bock, Ekaterina Pomerantseva

**Affiliations:** ^1^ Department of Materials Science and Engineering, Drexel University, Philadelphia, PA, United States; ^2^ Department of Materials Science and Chemical Engineering, Stony Brook University, Stony Brook, NY, United States; ^3^ Institute for Electrochemically Stored Energy, Stony Brook University, Stony Brook, NY, United States; ^4^ Brookhaven National Laboratory, Interdisciplinary Science Department, Upton, NY, United States; ^5^ Energy and Photon Sciences Directorate, National Synchrotron Light Source II, Brookhaven National Laboratory, Upton, NY, United States; ^6^ Department of Chemistry, Stony Brook University, Stony Brook, NY, United States

**Keywords:** MoO_3_, dopamine derived carbon, electronic conductivity improvement, aqueous energy storage, Zn-ion batteries

## Abstract

A capacitance increase phenomenon is observed for MoO_3_ electrodes synthesized *via* a sol-gel process in the presence of dopamine hydrochloride (Dopa HCl) as compared to α-MoO_3_ electrodes in 5M ZnCl_2_ aqueous electrolyte. The synthesis approach is based on a hydrogen peroxide-initiated sol-gel reaction to which the Dopa HCl is added. The powder precursor (Dopa)_x_MoO_y_, is isolated from the metastable gel using freeze-drying. Hydrothermal treatment (HT) of the precursor results in the formation of MoO_3_ accompanied by carbonization of the organic molecules; designated as HT-MoO_3_/C. HT of the precipitate formed in the absence of dopamine in the reaction produced α-MoO_3_, which was used as a reference material in this study (α-MoO_3_-ref). Scanning electron microscopy (SEM) images show a nanobelt morphology for both HT-MoO_3_/C and α-MoO_3_-ref powders, but with distinct differences in the shape of the nanobelts. The presence of carbonaceous content in the structure of HT-MoO_3_/C is confirmed by FTIR and Raman spectroscopy measurements. X-ray diffraction (XRD) and Rietveld refinement analysis demonstrate the presence of α-MoO_3_ and h-MoO_3_ phases in the structure of HT-MoO_3_/C. The increased specific capacitance delivered by the HT-MoO_3_/C electrode as compared to the α-MoO_3_-ref electrode in 5M ZnCl_2_ electrolyte in a −0.25–0.70 V vs. Ag/AgCl potential window triggered a more detailed study in an expanded potential window. In the 5M ZnCl_2_ electrolyte at a scan rate of 2 mV s^−1^, the HT-MoO_3_/C electrode shows a second cycle capacitance of 347.6 F g^−1^. The higher electrochemical performance of the HT-MoO_3_/C electrode can be attributed to the presence of carbon in its structure, which can facilitate electron transport. Our study provides a new route for further development of metal oxides for energy storage applications.

## Introduction

The structural abundance and rich chemical compositions of transition metals nanostructures have enabled them to play a key role in advancing energy storage technology (Pomerantseva et al., 2019; Liu et al., 2018). Transition metal oxides (TMOs) are among the highly redox active materials that are widely investigated. The use of oxides containing transition metal ions in high oxidation states as electrodes is an effective strategy to increase specific capacity. Orthorhombic molybdenum trioxide (α-MoO_3_) with a layered crystal structure has been recognized as one of the promising electrode materials for energy storage (Pomerantseva et al., 2019; Xie et al., 2020). In the synthesis of MoO_3_, various reaction parameters such as the molybdenum precursor, solvent medium, reaction time, and temperature allow for control over the morphology of the product (Pomerantseva et al., 2019; Mendez-Vivar et al., 1990), with various MoO_3_ nanostructures such as nanoparticles (Rajiv Chandar et al., 2020), nanowires (Joseph et al., 2019; Yu et al., 2020; Zhou et al., 2010), nanopapers (Yao et al., 2016; Zhao et al., 2020), and nanobelts (Wang et al., 2005; Li and Liu, 2013; Jiang et al., 2013) being reported. To improve the electrochemical properties of MoO_3_, strategies such as introducing oxygen vacancies (Kim et al., 2017), tuning the interlayer spacing (Yu et al., 2020), and incorporating conductive materials (Liu et al., 2013) have been proposed. An important feature of the orthorhombic MoO_3_, which makes many of the structural modifications possible, is the fact that the α-MoO_3_ structure is composed of MoO_6_ octahedra bilayers stacked along the *b-axis* held by weak van der Waals interactions (Supplementary Figure S1) (Tagaya et al., 1994; Yu et al., 2020). The layered structural network allows for the modification of interlayer chemistry *via*, for example, a chemical preintercalation synthesis approach (Mai et al., 2007; Mai et al., 2008; Dong et al., 2015). Compared to the prisitine α-MoO_3_, pre-lithiated (Mai et al., 2007; Mai et al., 2008) and pre-sodiated (Dong et al., 2015) α-MoO_3_ electrodes exhibited improved cycling stability and rate capability in non-aqueous Li-ion and Na-ion energy storage systems, respectively.

Energy storage systems with aqueous electrolytes have gained attention because of their low cost, safety, high ion conductivity, and ease of fabrication (Huang et al., 2019; Xie et al., 2020). Due to the layered structure, α-MoO_3_ represents a promising intercalation electrode in aqueous energy storage systems with various charge-carrying ions, such as Li^+^ (Tang et al., 2012; Jiang et al., 2013; Li and Liu, 2013; Yao et al., 2016; Zhao et al., 2020), Na^+^ (Liu et al., 2014; Elkholy et al., 2021), K^+^ (Liu et al., 2013), and Zn^2+^ (He et al., 2019; Wang et al., 2021) ions. The initial specific capacity delivered by α-MoO_3_ electrodes can be increased by expanding the potential window while remaining within the electrochemical water stability region (Elkholy et al., 2021). It was found that MoO_3_ electrodes suffer from capacity decay during charge/discharge arising as a result of the dissolution of Mo in aqueous electrolytes (Zhou et al., 2010; Elkholy et al., 2021; Wang et al., 2021). Another common challenge with MoO_3_ electrodes is the irreversible insertion of ions during the first discharge cycle, which greatly affects capacity (Li et al., 2006; Chen et al., 2010; Zhou et al., 2010; Lahan and Das, 2019). Additionally, α-MoO_3_ has a low intrinsic electronic conductivity (de Castro et al., 2017), which leads to structural degradation during cycling because of the hyperpolarization effect (Xiao et al., 2014; He et al., 2019; Shan et al., 2019; Elkholy et al., 2021). The electronic conductivity can be improved by integrating MoO_3_ with carbon and introducing a stable oxide/carbon heterointerface, similar to the vanadium and titanium oxide systems (Clites et al., 2020; Barim et al., 2021). Interestingly, the same strategy was shown to also result in slowing the capacity decay over cycling (Clites et al., 2020), which is highly desirable for α-MoO_3_ electrodes.

A tight oxide/carbon heterointerface can be enabled through the integration of the oxide and carbon precursors in a single reaction mixture. Dopamine hydrochloride (Dopa HCl) is established as an attractive carbon precursor due to its solubility in water enabling compatibility with aqueous-based synthesis approaches, its ability to bind to a wide range of organic and inorganic surfaces, and its efficient carbonization during heat treatment processes (Lee et al., 2007; Li et al., 2013; Yu et al., 2014; Lee et al., 2019). Incorporation of excess Dopa HCl into a sol-gel synthesis of molybdenum oxide, followed by hydrothermal treatment and/or annealing, resulted in the formation of hierarchically structured MoO_2_/C spheres (Norouzi et al., 2022). It was found that dopamine carbonization is accompanied by molybdenum reduction and resulted in MoO_2_ nanoplatelets distributed and confined on the surface of a dopamine-derived carbon matrix. Alternatively, the limited Dopa HCl concentration in a similar synthesis process with vanadium oxide produced small fractions of carbon in the interlayer region of the forming bilayered vanadium oxide phase. The product exhibited high vanadium oxidation state and enhanced charge storage properties in non-aqueous Li-ion cells (Clites et al., 2020). However, no reports on maintaining the high oxidation state of Mo in α-MoO_3_/carbon materials synthesized *via* integration with Dopa HCl exist. The electrochemical charge storage properties of such electrodes in aqueous energy storage systems remain unknown.

In this work, we present a new material, HT-MoO_3_/C, prepared by addition of a limited amount of Dopa HCl (molar ratio of Dopa: Mo = 1:5) into a sol-gel based synthesis, which was initiated by the reaction between hydrogen peroxide and Mo powder in aqueous media followed by hydrothermal treatment. A reference material, α-MoO_3_-ref, was prepared following the same procedure but without dopamine addition. Using XANES spectra analysis, the oxidation state of Mo in both HT-MoO_3_/C and α-MoO_3_-ref was determined to be close to + 6.0. Rietveld structure refinement of the XRD patterns revealed that molybdenum oxide in the HT-MoO_3_/C sample was a combination of α-MoO_3_ (93 wt%) and hexagonal MoO_3_ (h-MoO_3_, 7 wt%) phases, while SEM imaging showed uniform morphology consisting of nanobelt particles. Dopa carbonization was confirmed by Raman spectroscopy. We used aqueous cells with an electrolyte containing Zn^2+^ ions (5M ZnCl_2_) to evaluate electrochemical charge storage properties due to the interest of using aqueous Zn-ion batteries for grid-scale applications. Zn-ion batteries offer a low cost, safe and long lasting solution. Also, intercalation chemistry of divalent cations (Zn^2+^) is accompanied by multiple electron transfer leading to a power density advantage, and utilization of Zn metal as the negative electrodes offers an energy density advantage. We show that HT-MoO_3_/C electrodes outperform α-MoO_3_-ref electrodes in a −0.25–0.70 V vs. Ag/AgCl potential window. Improvements in specific capacitance are attributed to the structural modification induced by carbon incorporation. Electrochemical cycling of HT-MoO_3_/C electrodes in the expanded potential window of −0.85–1.00 V vs. Ag/AgCl demonstrated a second cycle specific capacitance of 347.6 F g^−1^ at a sweep rate of 2 mV s^−1^. We believe that our facile synthesis strategy holds potential in achieving materials characteristics that improve electrochemical properties of MoO_3_-based electrodes.

## Experimental Methods

### Synthesis of (Dopa)_x_MoO_y_, HT-MoO_3_/C, α-MoO_3_

A chemical preintercalation approach was used for the synthesis of dopamine preintercalated molybdenum oxide where metallic Mo was dissolved in water by the addition of hydrogen peroxide *via* a modified sol-gel process (Norouzi et al., 2022). First, dopamine hydrochloride ((HO)_2_C_6_H_3_CH_2_CH_2_NH_2_ HCl or Dopa HCl, Alfa Aesar) was dissolved in 2 ml of deionized water, followed by the addition of the stoichiometric amount of metallic Mo powder (Alfa Aesar) to the solution. The molar ratio of Mo:Dopa was kept at 5:1, similar to the previous study where a V_2_O_5_:Dopa molar ratio of 5:1 was used (Clites et al., 2020). Hydrogen peroxide (30 wt% H_2_O_2_, Alfa Aesar) was added dropwise until all Mo was dissolved; then the temperature of the reaction was set to 60°C. The solution viscosity increased after a few hours of heating, and the transparent solution was removed from the heat and kept in the fume hood where the cooling solution formed a gel after a few minutes. The gelled precursor was kept in the freezer at −20°C overnight, followed by freeze-drying (0.001 mbar, −84°C Freezone, Labconco), where the interlayer water was removed and the precursor was produced in the form of the powder. The precursor is denoted as (Dopa)_x_MoO_y_. The synthesis was repeated in the absence of dopamine, and a yellow precipitate was formed and filtered. The Mo:Dopa ratio of 5:1 warranted that the layered structure of MoO_3_ remained intact and the Mo maintained its high oxidation state. This is unlike our previous study where Mo:Dopa was kept at 1:1 and the simultaneous carbonization of polydopamine and reduction of oxygen saturated Mo species in the precursor to Mo^+4^ during hydrothermal treatment resulted in the formation of hierarchical structures of MoO_2_ and dopamine-derived carbon (Norouzi et al., 2022).

For carbonization, 300 mg of the (Dopa)_x_MoO_y_ precursor was placed into 12 ml of water in a 23 ml Teflon-lined autoclave (PARR, Acid Digest Vessel 23 ml) and kept at 180°C for 24 h. The hydrothermally treated product, denoted as HT-MoO_3_/C, was filtered, washed with deionized water, and dried at 105°C in air. HT-MoO_3_/C had a light blue color. The precipitate collected from the synthesis without dopamine was hydrothermally treated under identical conditions, and the white product was later characterized as α-MoO_3_ (called α-MoO_3_-ref), as it has been similarly reported in other literature (Mendoza-Sánchez et al., 2013).

### Materials Characterization

Scanning electron microscopy (SEM) was utilized to characterize particles morphology using a Zeiss Supra 50 VP instrument (Germany). X-ray diffraction (XRD) measurements were used for phase compositions characterization. XRD patterns were collected in a 2θ range of 4°–60° with a step size of 0.02° using a Rigaku SmartLab X-ray diffractometer (Japan) with Cu Kα radiation. Thermogravimetric analysis (TGA) was performed using a TA Instruments Q50 (TA Instruments, USA) and was conducted under air from room temperature to 700°C. Fourier-transform infrared spectroscopy (FTIR) and Raman spectroscopy were used to gather further details on the chemical composition of the synthesized materials. FTIR spectra were collected from 500 to 4,000 cm^−1^ using a Nicolet 6700 FTIR spectrometer. Raman spectra were obtained from 100 to 2,500 cm^−1^ using a Renishaw in *via* Raman microscope (Renishaw, United Kingdom) with a 514 nm Ar-ion laser. Transmission electron microscopy (TEM), high-resolution TEM (HRTEM) and high-angle annular dark-field scanning TEM (HAADF-STEM), selected area electron diffraction (SAED) and energy dispersive X-ray spectroscopy (EDS) data were collected using a JEOL 2100F operated at 200 kV located at the Center for Functional Nanomaterials at Brookhaven National Laboratory. Samples were sonicated and suspended in ethanol, and then drop cast on carbon supported TEM grids. Mo K-edge fluorescence X-ray absorption spectroscopy (XAS) data were collected at the National Synchrotron Light Source II (NSLS-II) beamline 8-ID, Inner Shell Spectroscopy, at Brookhaven National Laboratory. The synthesized materials were mixed with boron nitride (BN) for transmission XAS measurements. Samples were measured with a metal foil reference simultaneously for correct energy alignment of individual spectra during data analysis. The collected spectra were background subtracted, aligned, and normalized using the Athena software package. (Ravel and Newville, 2005). Linear combination fitting (LCF) was completed using spectra for two crystalline end members, MoO_2_ and MoO_3_, as reference materials to estimate the Mo oxidation state in synthesized materials. X-ray diffraction data for structure refinement were collected using a Rigaku SmartLab Diffractometer. Rietveld refinements were performed on the collected patterns using GSASII. (Toby and Von Dreele, 2013).

### Electrochemical Testing

The electrodes cycled in a −0.25–0.70 V vs. Ag/AgCl potential window were prepared by mixing an 80:15:5 by weight mixture of active material, activated carbon (YP-50, Kuraray Coal™) as a conducting agent, and poly (tetrafluoroethylene) binder (PTFE, Sigma-Aldrich), respectively, in a rotary mixer (FlackTek™) at 3,000 rpm. Ethanol was used to uniformly mix the components and achieve suitable wetting. HT-MoO_3_/C electrodes cycled in a −0.85–1.00 V vs. Ag/AgCl potential window were prepared using the same procedure but with a 70 (active material): 25 (YP-50): 5 (PTFE) weight ratio. The mixture was loaded on a carbon paper and rolled using a glass rod to achieve uniform sample loading with a desired thickness and mass. The cast was dried overnight in the fume hood. 3 mm electrodes with mass loadings of 0.04–0.06 mg were punched and used as the active material. Counter electrodes of 5 mm in diameter were prepared by mixing YP-50 carbon with ethanol until a homogeneous paste was formed, then rolling the paste using a glass rod on a cleaned glass surface.

The electrochemical performance of HT-MoO_3_/C and α-MoO_3_-ref electrodes were studied in an aqueous 5M ZnCl_2_ electrolyte in a 3-electrode Swagelok™ cell setup. All potentials in this work are reported with respect to the Ag/AgCl reference electrode. Cyclic voltammetry (CV) experiments were carried out using a Biologic™ potentiostat. CV curves were obtained by cycling electrodes in −0.25–0.70 V and −0.85–1.00 V potential windows with a scan rate of 1, 2, 5, 10, and 20 mV s^−1^.

## Results

**FIGURE 1:**
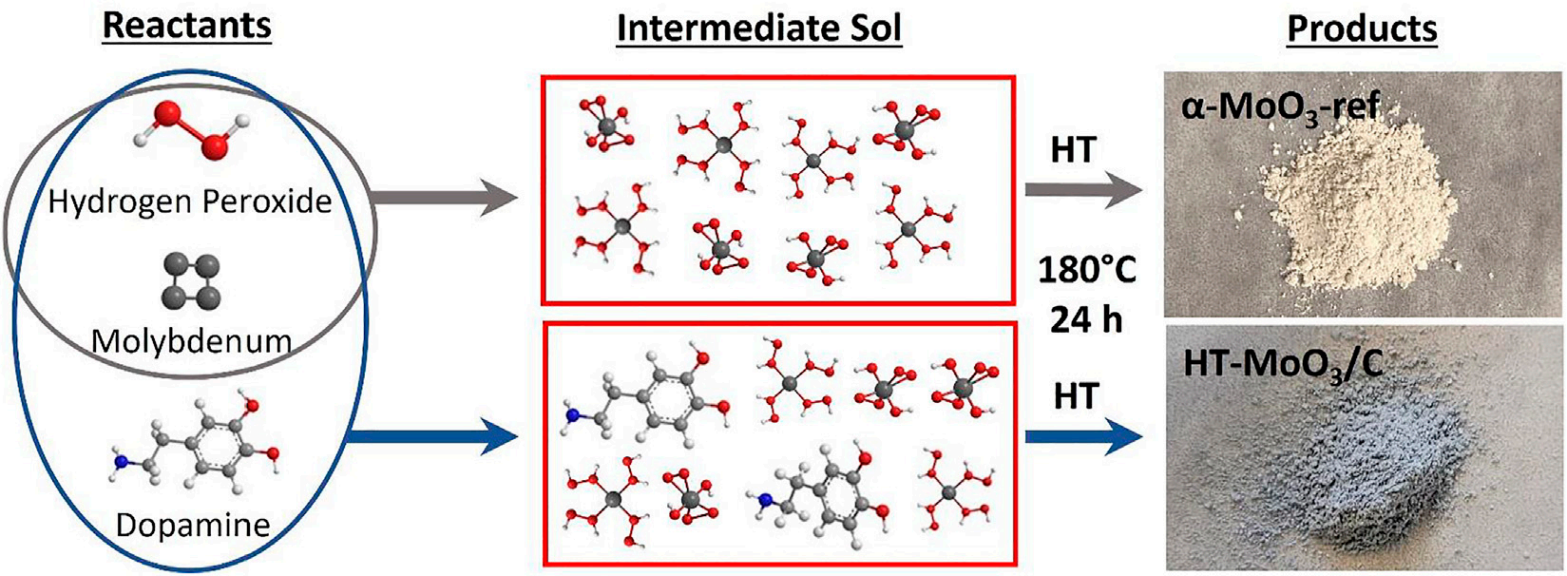
Schematic illustration of the synthesis process involving sol-gel reaction between metallic molybdenum and hydrogen peroxide with and without the presence of dopamine hydrochloride. The product of the sol-gel reaction was hydrothermally treated (HT) to produce white α-MoO_3_-ref and light-blue HT-MoO_3_/C powders.

The synthesis strategy used to integrate MoO_3_ with carbon is schematically shown in Figure 1. Metallic molybdenum is dissolved in an aqueous solution using hydrogen peroxide in the presence of a deficient amount of dopamine hydrochloride. The gel-like product of the sol-gel reaction is composed of dopamine preintercalated molybdenum oxide, and it is isolated as a powder after freeze-drying. The product, denoted as (Dopa)_x_MoO_y_, is used as a precursor for the hydrothermal synthesis and produced a powder of light blue color, called HT-MoO_3_/C. This synthesis is also performed without dopamine, in which the hydrothermal treatment of the yellow precipitate yields a white powder of MoO_3_ with orthorhombic crystal structure, also known as α-MoO_3_. The as-synthesized α-MoO_3_ is used as a reference (called α-MoO_3_-ref) to study the effect of the modified synthesis process on the structure and electrochemistry of MoO_3_ electrodes in aqueous energy storage systems.

**FIGURE 2:**
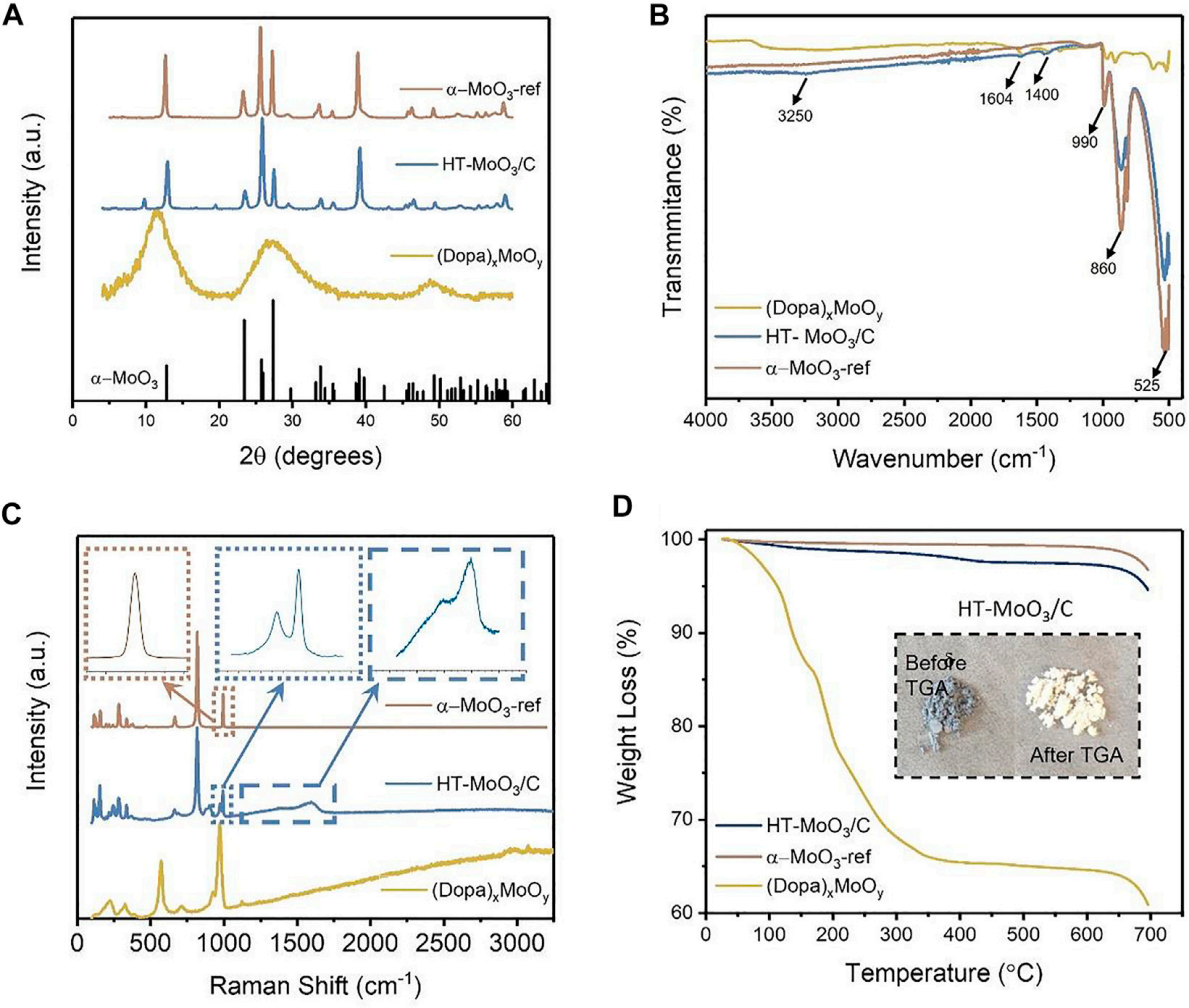
Structural and chemical characteristics of the (Dopa)_x_MoO_y_, HT-MoO_3_/C, and α-MoO_3_-ref: **(A)** XRD patterns, **(B)** FTIR spectra, **(C)** Raman spectra, and **(D)** TGA weight loss curves.

The XRD pattern of the (Dopa)_x_MoO_y_ precursor showed (00*l*) reflections with decreasing reflection intensities as the 2θ value increased, which is typical for layered structures without long-range order (Petkov et al., 2002) and is attributed to the preintercalation of dopamine molecules into the interlayer region of molybdenum oxide (Figure 2A). The XRD patterns of HT-MoO_3_/C and α-MoO_3_-ref both exhibit peaks that correspond to the orthorhombic α-MoO_3_ structure (JCPDS #05-0508) (Yu et al., 2020; Li and Liu, 2013; Xiao et al., 2015). However, the XRD pattern of HT-MoO_3_/C shows additional small peaks, with predominance of the peak at 9.9°2θ (Figure 2A). FTIR spectra of the (Dopa)_x_MoO_y_ precursor, its hydrothermally treated product, and reference α-MoO_3_ are shown in Figure 2B. The bands centered at 525, 860, and 990 cm^−1^ are attributed to the stretching vibrations of Mo-O (2), Mo-O (3), where the Mo atom is linked with three and two oxygen atoms and Mo-terminal oxygen, respectively (Yao et al., 2016; Mai et al., 2007; Qi et al., 2006). The presence of foreign species in the interlayer region of the precursor and its hydrothermally treated product was further confirmed by the signals in the 1,400–4,000 cm^−1^ range in the FTIR spectra (Qi et al., 2006). Weak signals at 1,400 and 1,604 cm^−1^ and a broad signal centered at 3,250 cm^−1^ are present in the spectra of both the (Dopa)_x_MoO_y_ precursor and the HT-MoO_3_/C and correspond to the interlayer dopamine and interlayer carbon, respectively (Mallinson et al., 2018). Raman spectroscopy measurements confirmed the presence of carbon in the composition of the hydrothermally treated (Dopa)_x_MoO_y_ precursor (Figure 2C), and therefore it is called HT-MoO_3_/C. The Raman spectra of HT-MoO_3_/C show two characteristic carbon bands between 1,360 and 1,630 cm^−1^, corresponding to the D and G bands of carbon. For HT-MoO_3_/C, the D band and G band are observed at 1,394 and 1,593 cm^−1^, with an I_D_/I_G_ ratio of 0.95. The bands in the 100–1,100 cm^−1^ region are characteristic of MoO_3_. The high-intensity peaks at 991, 818, 662, and 282 cm^−1^ correspond to MoO_3_ with some degree of surface oxidation (Zhao et al., 2020). The low-frequency peaks at 120 and 197 cm^−1^ are indicative of Mo-Mo bending (Yao et al., 2016; Yao et al., 2020). Thermal stability of the (Dopa)_x_MoO_y_ precursor, HT-MoO_3_/C, and MoO_3_ was evaluated *via* thermogravimetric analysis (TGA) from room temperature to 700°C in air, as shown in Figure 2D. The precursor (Dopa)_x_MoO_y_ shows the most pronounced weight loss starting with the loss of surface adsorbed water (15 wt%) in the temperature range of 50–150°C, followed by decomposition of interlayer dopamine molecules under air. The TGA curve collected for the (Dopa)_x_MoO_y_ precursor levels off at ∼ 600°C, which corresponds to a 20% weight loss and agrees with the amount of dopamine (Mo:Dopa, 5:1) used in the sol-gel synthesis of the precursor. The TGA curve collected for the HT-MoO_3_/C sample shows a weight loss of 3%, corresponding to the decomposition of dopamine-derived carbon starting at 280°C. The dopamine molecules in the interlayer region of the precursor are carbonized under the hydrothermal condition, and the more thermodynamically stable dopamine-derived carbon in HT-MoO_3_/C is decomposed at higher temperatures than dopamine molecules in the (Dopa)_x_MoO_y_ precursor. The inset in Figure 2D shows the photographs of the HT-MoO_3_/C sample at the beginning and at the end of the TGA experiment. The light blue color of the HT-MoO_3_/C powder changes to a white color, similar to the color of α-MoO_3_-ref powder (Figure 1), which could be attributed to the oxidation of molybdenum oxide under air.

**FIGURE 3:**
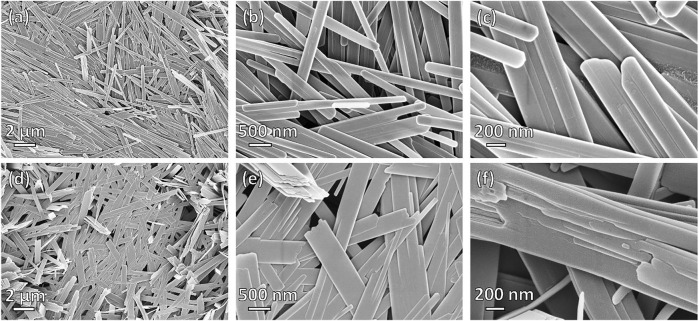
SEM images of the **(A–C)** α-MoO_3_-ref and **(D–F)** HT-MoO_3_/C nanobelts **(A, D)** Low- and **(B, C, E, F)** high-magnification images are shown.

Figure 3 shows the morphology of the reference α-MoO_3_-ref powder (Figures 3A–C) and the hydrothermally treated (Dopa)_x_MoO_y_ precursor, HT-MoO_3_/C (Figures 3D–F). The reference material crystallized in the form of nanobelts with rounded ends and a relatively smooth surface (Figures 3A–C). The material synthesized with dopamine also exhibited nanobelt morphology (Figures 3D–F), though different from the α-MoO_3_-ref nanobelts. The HT-MoO_3_/C nanobelts have sharper edges and they appear wider than the α-MoO_3_-ref nanobelts. Additionally, the ends of some HT-MoO_3_/C nanobelts are rippled and fragmentation in the layered structure can be seen on their surface (Figures 3E,F). These structural deformations could be caused by interactions between the interlayer dopamine and MoO_3_ layers under hydrothermal treatment conditions. A similar morphological modification was observed in the case of chemical preintercalation synthesis of molybdenum oxide in a system enriched with dopamine, where annealing of the hydrothermally treated MoO_2_/C powder led to the splitting of MoO_2_ nanoplatelets on the surface of the dopamine-derived carbon spheres (Norouzi et al., 2022). Additional SEM images of the HT-MoO_3_/C nanobelts that demonstrate morphological features different from the α-MoO_3_-ref nanobelts are shown in Supplementary Figure S2.

**FIGURE 4:**
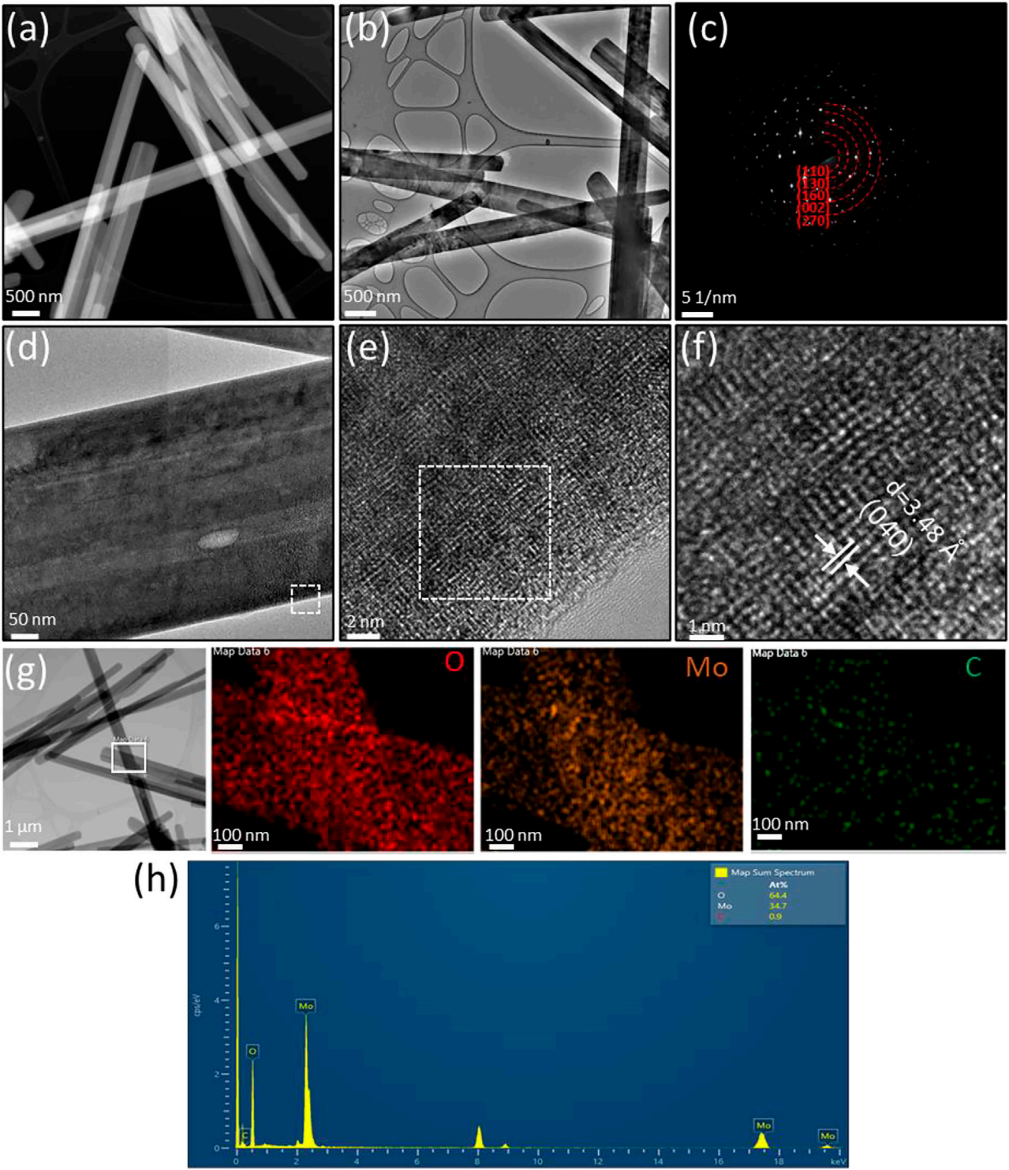
Structural characterization of α-MoO_3_-ref. **(A)** HAADF-STEM image. **(B)** TEM image. **(C)** SAED pattern **(D–F)** HRTEM image of the area marked by the white box in **(D)**. **(G–H)** TEM-EDS mapping and spectroscopy of the area marked by the white box in **(G)**.

**FIGURE 5:**
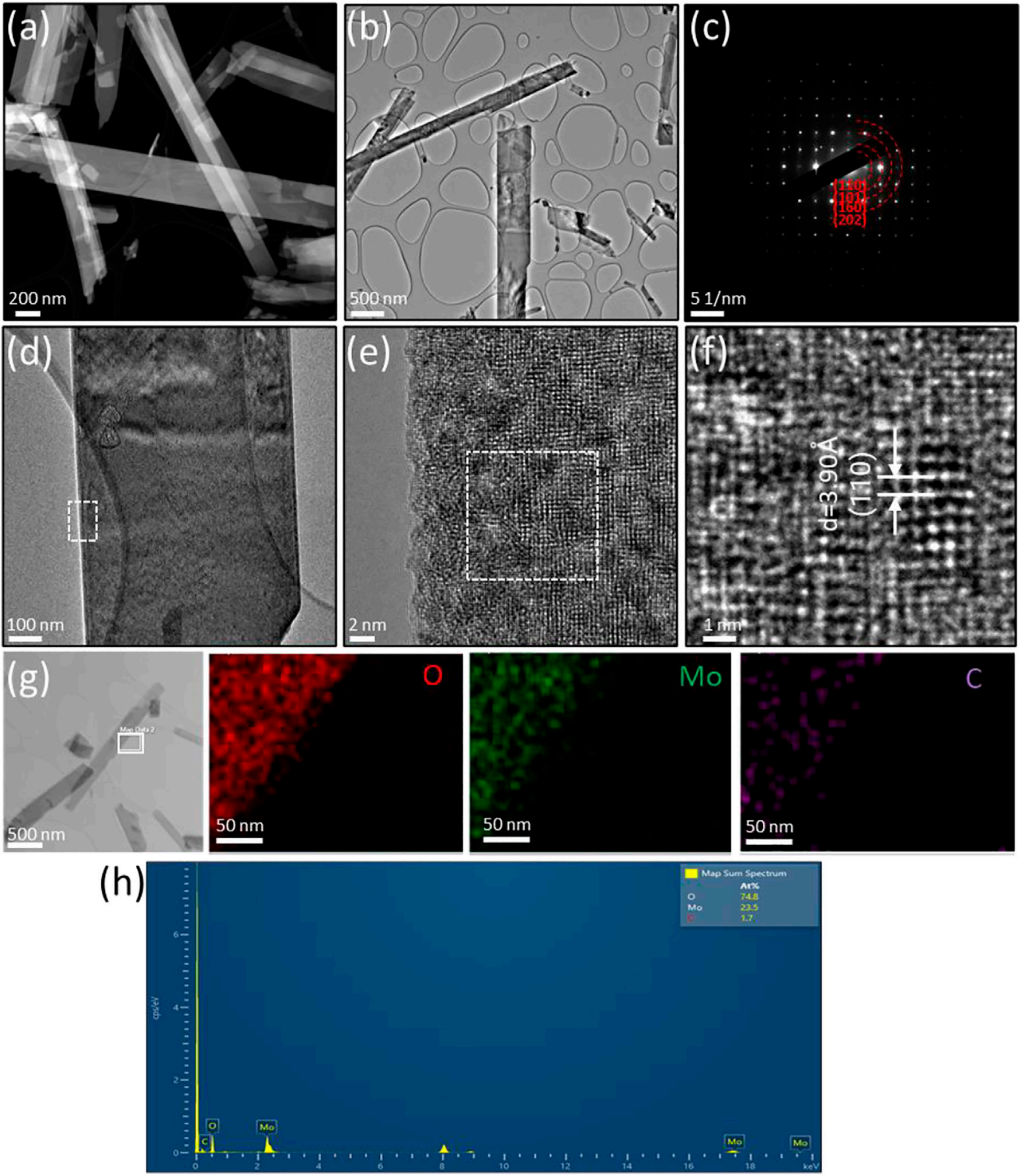
Structural characterization of HT-MoO_3_/C. **(A)** HAADF-STEM image. **(B)** TEM image. **(C)** SAED pattern **(D–F)** HRTEM image of the area marked by the white box in **(D)**. **(G–H)** TEM-EDS mapping and spectroscopy of the area marked by the white box in **(G)**.

The morphologies, structures and compositions of α-MoO_3_-ref (Figure 4) and HT-MoO_3_/C (Figure 5) are further compared by TEM-EDS images. The HADDF-STEM and TEM images of α-MoO_3_-ref shows uniform nanobelt morphologies with width around 400 nm and length around 5 µm (Figures 4A,B). The SAED patterns of α-MoO_3_-ref (Figure 4C) indicate polycrystalline structures with (110) (130), (160), (002), and (270) phases, that match the patterns from α-MoO_3_ structure (JCPDS #05-0508). Figures 4D–F shows the HRTEM images of a single α-MoO_3_-ref nanobelt, that indicates a crystalline structure both at the edge and center of nanobelt. The lattice fringe with a d-spacing measured at 3.48 Å can be indexed to the (040) plane. The TEM-EDS mapping images and spectroscopy show Mo and O elements distributed uniformly with a small amount of carbon (0.9 atomic %) (Figures 4G,H). This small amount of carbon can be adventitious carbon where a thin layer of carbonaceous material is usually found on the surface of most samples as the sample was exposed to air. Compared to α-MoO_3_-ref, the HADDF-STEM and TEM images of the HT-MoO_3_/C nanobelts shows similar morphology but with some broken pieces (Figures 5A,B). The SAED patterns of HT-MoO_3_/C (Figure 5C) indicate polycrystalline structures with (110), (010), (160), and (202) phases and also match the patterns from α-MoO_3_ structure (JCPDS #05-0508). Figures 5D–F shows crystalline structure of at the edge and center of a single HT-MoO_3_/C nanobelt from the HRTEM images, and no amorphous carbon phases were observed. The presence of only 3 wt% of carbon in HT-MoO_3_/C nanobelts based on the TGA curve makes the observation of the carbon in a local environment unlikely. Further, as discussed above, the simultaneous crystallization of the oxide phase and carbonization of dopamine under hydrothermal treatment conditions has resulted in a tightly integrated MoO_3_/C structure, which is different from the carbon coated shell structure as we reported previously (Norouzi et al., 2022). The lattice fringe with a d-spacing measured at 3.90 Å can be indexed to the (110) plane. The TEM-EDS mapping images and spectroscopy show Mo and O elements distributed uniformly with tiny carbon (1.7 atomic %) (Figures 5G,H). Compared to the α-MoO_3_-ref, there are 0.8 atomic % amount of carbon increased for HT-MoO_3_/C. This small amount of carbon difference could be a result of the integrated carbon or adventitious carbon. Additionally, TEM images provide local material characterization. Previous report presented a similar synthesis approach for the preparation of δ-V_2_O_5_/C integrated structures and showed that carbon forms intermittently within the oxide structure (Clites et al., 2020). The same phenomenon can be happening in case of molybdenum oxide system. The small amount of carbon combined with its localized formation make it challenging to clearly demonstrate its presence using TEM characterization. Raman spectrum of the HT-MoO_3_/C, however, convincingly indicates carbon in the chemical composition of the synthesized material.

**FIGURE 6:**
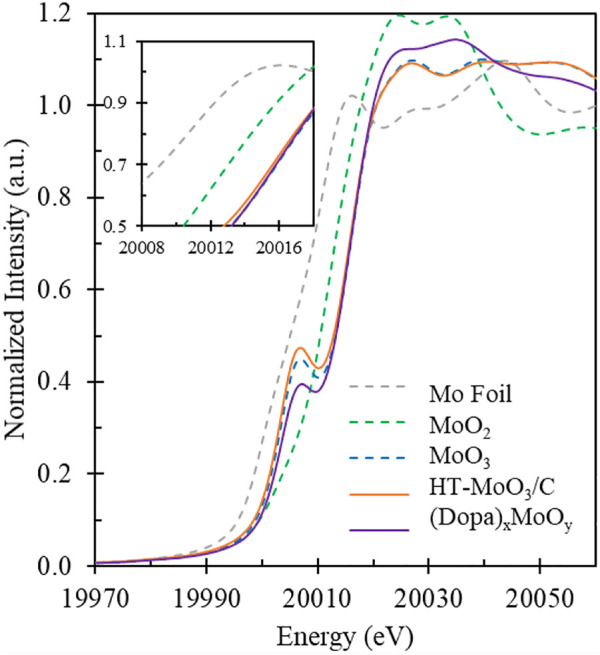
Mo K-edge XANES spectra of (Dopa)xMoO_3_ precursor and HT-MoO_3_/C samples next to Mo foil, MoO_2_ and MoO_3_ standards.

To study the localized coordination environment of Mo atoms in samples, synchrotron based X-ray absorption near-edge spectra (XANES) measurements were conducted. The Mo K-edge XANES spectra of the HT-MoO_3_/C and (Dopa)_x_MoO_3_ precursor as well as the data for the references are shown in Figure 6. Linear combination fitting (LCF) was performed to obtain an estimate of the oxidation state of the Mo metal center. The detailed fitting results are displayed in Table 1. From the LCF analysis, the Mo oxidation state in the (Dopa)_x_MoO_3_ precursor is + 5.6. Hydrothermal treatment of (Dopa)_x_MoO_3_, however, appears to oxidize molybdenum leading to the oxidation state of + 6.0 in HT-MoO_3_/C.

**TABLE 1:** LCF results of XANES spectra.

Sample	R factor	Reduced chi	MoO_2_	MoO_3_	Ox. State	Error (%)
HT-MoO_3_/C	0.00136	0.000166	0.7% (1.5%)	99.2% (1.5%)	6.0	2.2
(Dopa)_x_MoO_y_	0.00506	0.000763	20.2% (3.2%)	79.8% (3.2%)	5.6	4.6

**FIGURE 7:**
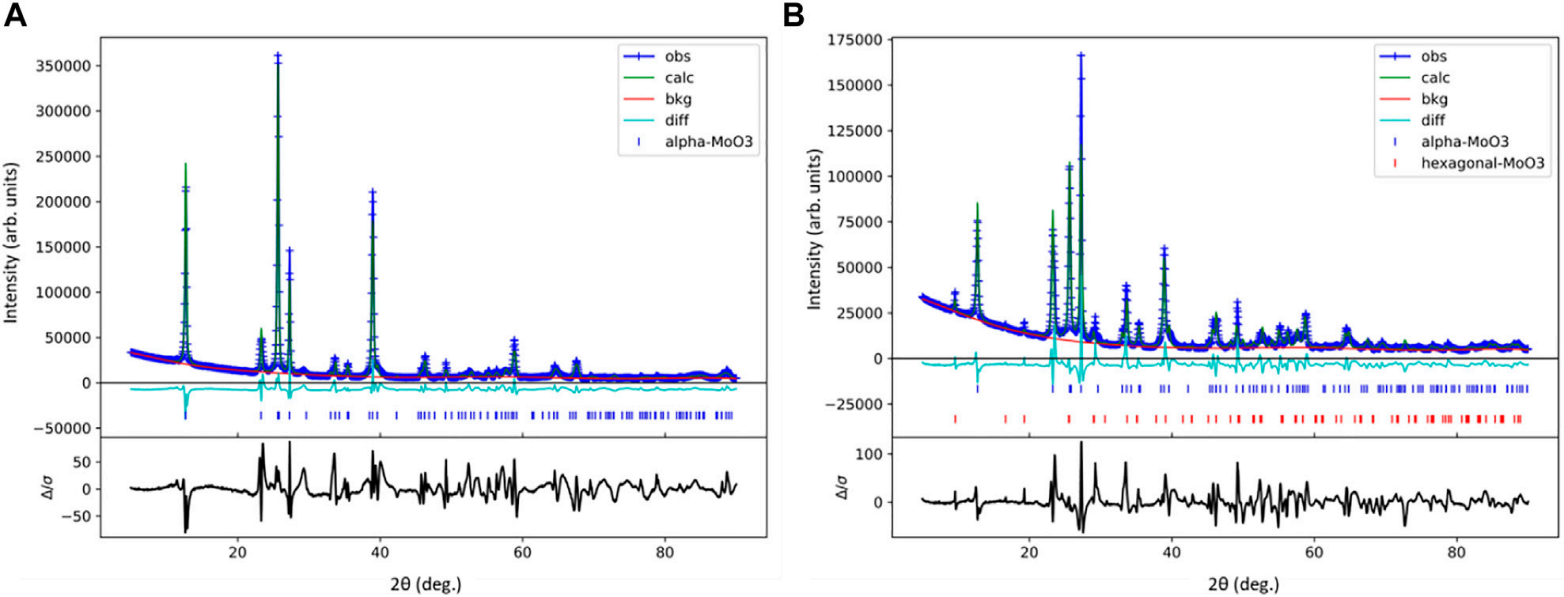
Rietveld refinement of the XRD patterns collected for **(A)** α-MoO_3_-ref and **(B)** HT-MoO_3_/C samples.

To better understand the differences between the α-MoO_3_-ref and HT-MoO_3_/C materials, Rietveld refinements were performed on respective XRD patterns (Figure 7). Any preferred crystallographic orientation present in the collected XRD data was corrected for using a generalized spherical harmonic model. (Von Dreele, 1997). The refinement parameters are shown in Table 2. The refinement results revealed that the HT-MoO_3_/C material is composed of both orthorhombic α-MoO_3_ (93 wt%) and hexagonal MoO_3_ (h-MoO_3_, 7 wt%) (Lunk et al., 2010) components. The schematic illustration of the h-MoO_3_ structure is shown in Supplementary Figure S1B in the Supporting Information. The h-MoO_3_ phase has been reported to form in systems containing organic molecules (Zakharova et al., 2018), and it usually crystallizes in the form of hexagonal rods (Lunk et al., 2010; Zakharova et al., 2018). However, detailed analysis of the SEM images of HT-MoO_3_/C revealed a rather homogenous nanobelt morphology without the presence of particles of different shapes. The slightly increased unit cell parameters of the dominating α-MoO_3_ component of the HT-MoO_3_/C sample, as compared to the unit cell parameters calculated for the α-MoO_3_-ref sample (Table 2) may indicate a small fraction of oxygen vacancies forming in the material structure, indicated by the light blue sample color (Kim et al., 2017). While this assumption is not confirmed by the results of XANES spectra analysis, showing + 6.0 oxidation state of Mo centers in HT-MoO_3_/C (Figure 6 and Table 1), given the method error and relatively small change in unit cell parameters revealed by Rietveld refinement, we suggest that the fraction of oxygen vacancies is relatively low. Another factor that attests to the possibility of oxygen vacancies formation is the splitting of the band at ∼ 1,000 cm^−1^ in the Raman spectrum of HT-MoO_3_/C powder, while no such splitting is observed in the Raman spectrum of α-MoO_3_-ref (Figure 2C). A similar effect of oxygen vacancies formation on Raman spectra was reported for plasma etched α-MoO_3_ (Zhang et al., 2018). The possibility and control of oxygen vacancies formation in our synthesis approach needs to be investigated further.

**TABLE 2:** Rietveld refinement parameters of MoO_3_ and HT-MoO_3_/C samples.

Sample		Space group	A (Å)	B (Å)	C (Å)	%R_wp_	Crystallite size (nm)	wt%

α-MoO_3_-ref		*Pnma*	3.9620 (8)	13.864 (1)	3.7003 (5)	12.85	29.5 (2)	100
HT-MoO_3_/C	α-MoO_3_	*Pnma*	3.9655 (5)	13.877 (1)	3.7056 (4)	13.74	41.8 (1)	93 (2)
	h-MoO_3_	*P*6_3_/*m*	10.61 (3)	10.61 (3)	3.772 (2)		56 (1)	7 (1)

**FIGURE 8:**
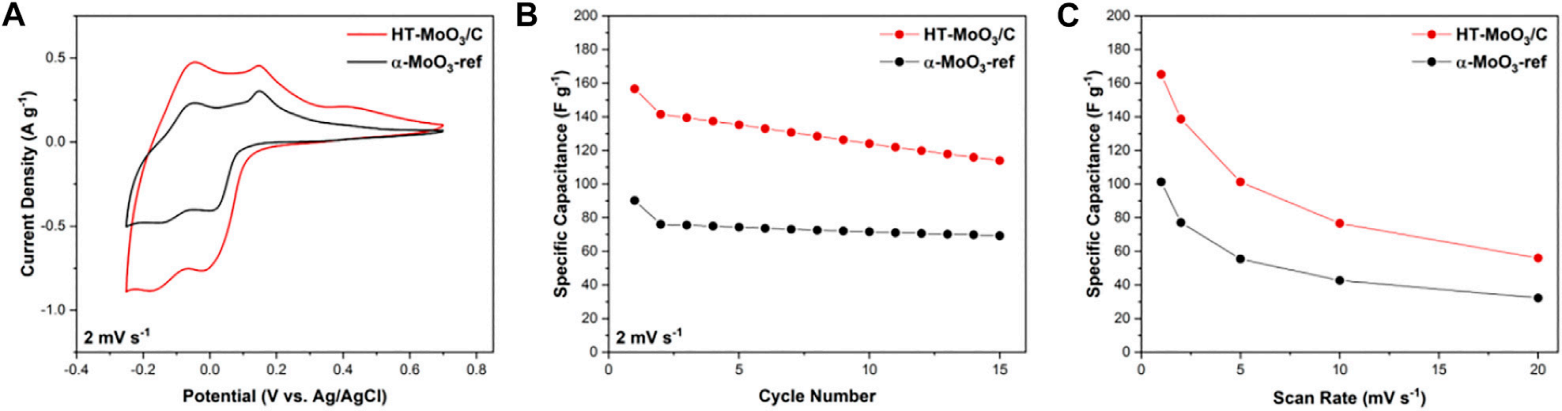
Electrochemical charge storage study of HT-MoO_3_/C and α-MoO_3_-ref electrodes in cells containing 5M ZnCl_2_ aqueous electrolyte in a potential window from −0.25 to 0.70 V: **(A)** second cycle CV curves, **(B)** cycling stability at a scan rate of 2 mV s^−1^, and **(C)** rate capability at increasing scan rates (1, 2, 5, 10, and 20 mV s^−1^).

The electrochemical performance of the HT-MoO_3_/C electrodes in comparison to the α-MoO_3_-ref electrodes was evaluated in an aqueous system using Swagelok T-cells. The second cycle cyclic voltammetry (CV) profiles for cells cycled in 5M ZnCl_2_ electrolyte in a potential window of −0.25–0.70 V are shown in Figure 8A (the corresponding first cycle CV curves are included in Supplementary Figure S3). The first to second cycle irreversibility is associated with the trapping of electrochemically cycled ions in the crystal structure, which has been previously reported to cause an unrecoverable structure transformation of α-MoO_3_ (Zhou et al., 2010; Li et al., 2006; Chen et al., 2010). The reversible redox peaks appearing at −0.16 V on discharge (with a corresponding peak at −0.05 V on charge) and −0.02 V on discharge (with a corresponding peak at 0.16 V on charge) are usually indicative of reversible intercalation type behavior. The second-cycle capacitance delivered by the HT-MoO_3_/C electrode (141.4 F g^−1^) is nearly 2-fold higher than that of the reference α-MoO_3_ electrode (76.1 F g^−1^). The increased capacitance could be attributed to the more efficient electron transport in the structure of the HT-MoO_3_/C active material due to the carbon presence. The increase in electronic conductivity of the HT-MoO_3_/C as compared to α-MoO_3_-ref was confirmed by four-probe conductivity measurements (Supplementary Table S1). Additionally, oxygen vacancies, if present, could facilitate ion transport through the crystal lattice of the α-MoO_3_ structure (Kim et al., 2017). The HT-MoO_3_/C electrode outperformed the α-MoO_3_-ref electrode at each of the following cycles for 15 cycles (Figure 8B). However, it shows a faster capacitance fading. The capacitance of the HT-MoO_3_/C electrode dropped to 114.0 F g^−1^ on the 15th cycle (19.4% decrease compared to the 2nd cycle capacitance), while the reference α-MoO_3_ electrode exhibited a capacitance of 69.3 F g^−1^ on the 15th cycle (8.9% decrease compared to the 2nd cycle capacitance). The capacitance fading of MoO_3_ electrodes in aqueous energy storage systems over extended cycling is attributed to the dissolution of molybdenum in water-based electrolytes (He et al., 2019). A more rapid degradation of the HT-MoO_3_/C electrode could be associated with oxygen vacancies, which may destabilize the structure of α-MoO_3_ and promote undesirable phase transformations and dissolution in the electrolyte. When cycled at increasing sweep rates, the HT-MoO_3_/C electrode consistently showed higher capacitance values compared to those delivered by the α-MoO_3_-ref electrode (Figure 8C), which could be ascribed to the improved charge transport enabled by the presence of carbon in the material structure and possible contributions of oxygen vacancies. The presence of oxygen vacancies, though, needs to be further confirmed. Our current results do not allow us to make a solid conclusion on the existence of oxygen vacancies in the structure of the HT-MoO_3_/C. Modification of the synthesis approach that would allow for control of the oxygen sublattice in α-MoO_3_ structure would be helpful and is currently the focus of our research.

**FIGURE 9:**
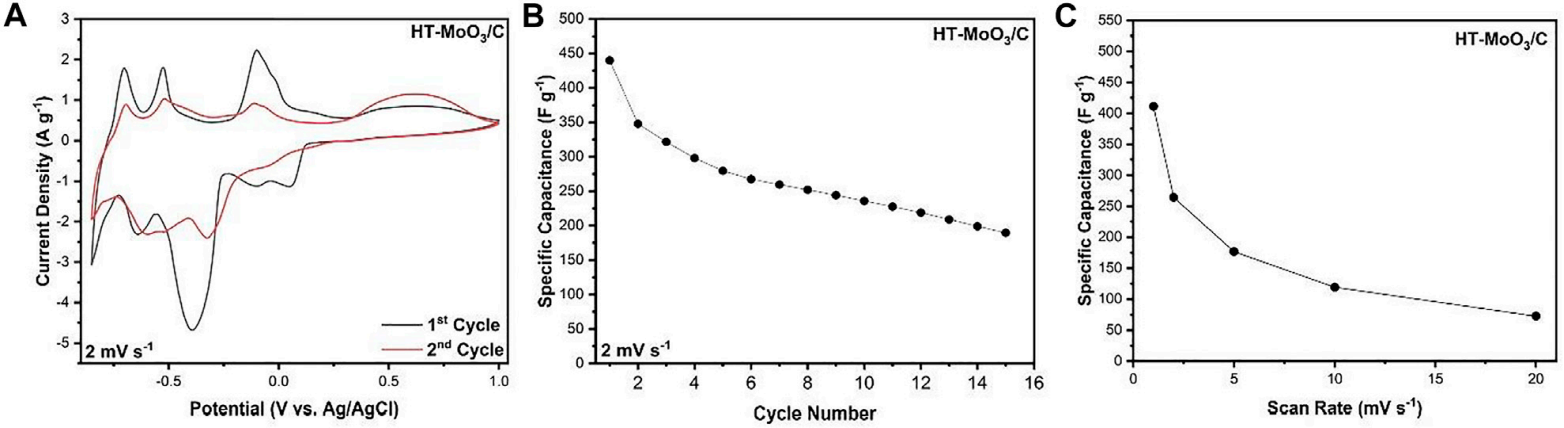
Electrochemical charge storage performance of the cells containing HT-MoO_3_/C electrodes in 5M ZnCl_2_ aqueous electrolyte in a potential window from −0.85 to 1.00 V. **(A)** First and second cycle CV curves at a scan rate of 2 mV s^−1^, **(B)** cycling stability at a scan rate of 2 mV s^−1^, and **(C)** rate capability at increasing scan rates (1, 2, 5, 10, and 20 mV s^−1^).

The advanced electrochemical charge storage properties exhibited by the HT-MoO_3_/C electrode in −0.25–0.70 V potential window triggered our interest to investigate its electrochemistry in the expanded potential window of −0.85–1.00 V, following a study demonstrating that the α-MoO_3_ structure showed redox activity at potentials closer to the electrochemical water stability window limits (Elkholy et al., 2021). The water electrolysis reaction was observed at potentials lower than −0.85 V, which can be attributed to the acidic condition of the zinc chloride solution. Figure 9A shows the CV curves of the HT-MoO_3_/C electrode without evidence of the features of the water-splitting reaction.

Further redox activity occurs upon expansion of the potential range. On the 1st cycle at 2 mV s^−1^, in addition to the peaks observed in the restricted window of −0.25–0.70 V, prominent discharge peaks are evident at −0.39 V, −0.63 V, and −0.79 V, with corresponding charge peaks at −0.10 V, −0.53 V, and −0.70 V respectively. Moreover, a broad peak centered at 0.57 V is witnessed during charging. This phenomenon is also observed when HT-MoO_3_/C is cycled in the narrow potential window. Therefore, it is believed that the electron transfer capability of the active material is facilitated, as suggested by the four-probe conductivity measurements (Supplementary Figure S4 and Supplementary Table S1). The 2nd cycle CV curve shows that all features from the 1st cycle are retained (Figure 9A), albeit with minor potential shifts and suppressed current densities possibly due to irreversible intercalation of a fraction of cycled ions within the crystal structure. The broad feature appearing towards the upper limit of the potential window is an exception, with greater charge storage occurring on cycle 2 in comparison to cycle 1. The HT-MoO_3_/C electrode exhibits a capacitance of 347.6 F g^−1^ on the second cycle. However, on further cycling, the capacitance of the electrode drops significantly, possibly due to the dissolution of MoO_3_ in the aqueous electrolyte (He et al., 2019). After 15 cycles, a capacitance of 189.4 F g^−1^ is retained, representing a 45.5% capacitance decay from the second cycle (Figure 9B). The CV profiles of HT-MoO_3_/C continuously cycled at 2 mV s^−1^ show a general diminishing of redox activity, but a broad redox couple, centered at –0.59 V during discharge cycles and –0.47 V during charge cycles, evolves (Supplementary Figure S5A). This reversible redox process persists when the HT-MoO_3_/C electrode is cycled at increasing scan rates up to 20 mV s^−1^ (Supplementary Figure S5B), although the overpotential between the peaks increases due to an increasing diffusion limitation with increasing scan rate. The HT-MoO_3_/C electrode delivers up to 72.8 F g^−1^ in capacitance at 20 mV s^−1^ (Figure 9C).

Our results are consistent with prior studies which showed that the electrode containing carbon in the layered structure showed improved electrochemical performance (Jiang et al., 2012; Wen et al., 2014; Wang et al., 2019; Clites et al., 2020). The further development of HT-MoO_3_/C electrodes for aqueous energy storage involves understanding carbon structure and its localization. This can be achieved through the modification of the synthesis to ensure the formation of a larger carbon fraction by either selecting alternative organic molecules as a precursor or utilizing solvothermal treatment in liquid organic solvents. Higher carbon content, if achieved, would also potentially lead to further improvements of charge transport and enhanced electrochemistry. Controllable oxygen vacancy formation, discussed above, can further facilitate ion transport by creating additional channels for ion diffusion through the Mo-O layers. Although, the optimum oxygen vacancy concentration needs to be found, as excessive anionic sublattice deficiency can lead to structural and electrochemical instabilities (Zhang et al., 2018). Electrochemical instability of HT-MoO_3_/C electrodes caused by dissolution in aqueous electrolytes could be mitigated by utilizing water-in-salt electrolytes (WISE), which can also result in increased capacitance due to the modification of the mechanism of charge storage in water-deficient systems (Wang et al., 2020; Wang et al., 2021).

## Conclusion

In this study, we present a new MoO_3_-based material that shows increased capacitance compared to a reference α-MoO_3_ electrode when cycled in aqueous 5M ZnCl_2_ electrolyte. The new material was synthesized *via* the incorporation of Dopa HCl into a sol-gel process initiated by a reaction between H_2_O_2_ and metallic Mo powder in water. The simultaneous crystallization of the oxide phase and carbonization of dopamine under hydrothermal treatment (HT) conditions has resulted in a tightly integrated oxide/carbon structure; therefore, the new material is called HT-MoO_3_/C. X-ray diffraction (XRD) and Rietveld refinement analysis revealed a 7 wt% admixture of h-MoO_3_ in the HT-MoO_3_/C structure, while phase composition was dominated by α-MoO_3_ (93 wt%). The presence of carbon was confirmed by Raman and FTIR spectroscopy measurements, as well as TGA. Interestingly, SEM images showed homogeneous nanobelt morphology, typical for α-MoO_3_, without signs of foreign phases. The integration of carbon into the MoO_3_ structure improved the electrochemical performance in aqueous Zn-ion electrochemical cells. Our results lay the foundation for further investigation of the HT-MoO_3_/C material structure, formation mechanism, and electrochemical performance.

## Data Availability

The raw data supporting the conclusion of this article will be made available by the authors, without undue reservation.
