# Supplementary material for: The Dopamine Assisted Synthesis of MoO3/Carbon Electrodes With Enhanced Capacitance in Aqueous Electrolyte
Source: Front Chem. 2022 Apr 19;10:873462. doi: 10.3389/fchem.2022.873462 (PMC9062078; doi:10.3389/fchem.2022.873462)
Supplement: Supplementary file 1 [file DataSheet1.PDF]

The dopamine assisted synthesis of MoO_3/carbon electrodes with enhanced capacitance in aqueous electrolyte

Nazgol Norouzi[1], Darrell Omo-Lamai[1], Farbod Alimohammadi[1], Timofey Averianov[1], Jason Kuang[2,3], Lei Wang[3,4], Eli Stavitski[6], Denis Leshchev[6], Kenneth J. Takeuchi[2,3,4,5], Esther S. Takeuchi[2,3,4,5], Amy C. Marschilok[2,3,4,5], David C. Bock[3,4], Ekaterina Pomerantseva[1*]

1. Department of Materials Science and Engineering, Drexel University, Philadelphia, PA 19104, USA
2. Department of Materials Science and Chemical Engineering, Stony Brook University, Stony Brook, NY 11794, USA
3. Institute for Electrochemically Stored Energy, Stony Brook University, Stony Brook, NY 11794, USA
4. Interdisciplinary Science Department, Brookhaven National Laboratory, Upton NY 11973, USA
5. Department of Chemistry, Stony Brook University, Stony Brook, NY 11794, USA
6. Energy and Photon Sciences Directorate, National Synchrotron Light Source II, Brookhaven National Laboratory, Upton NY 11973, USA

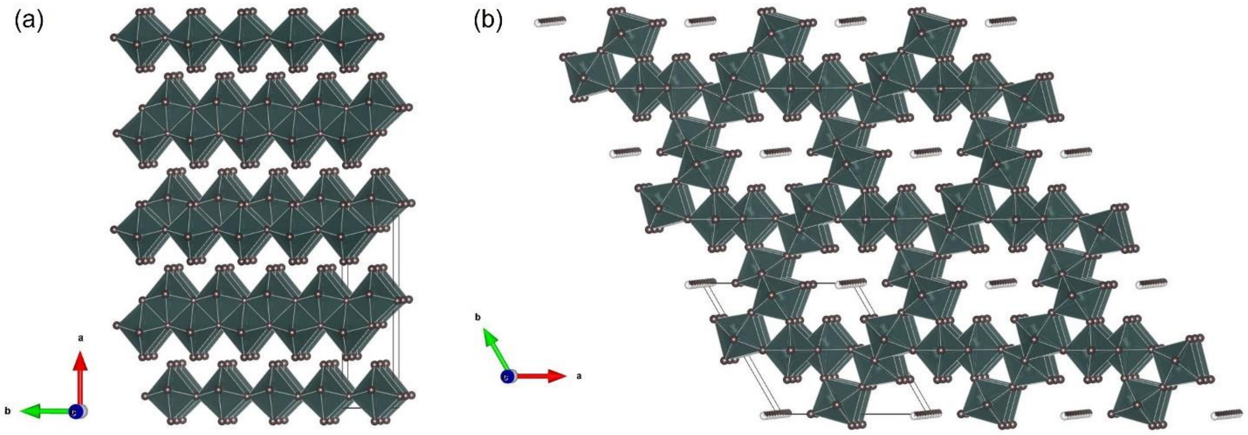

Figure S1. Schematic illustrations of the crystal structures of (a) α-MoO_3 and (b) hexagonal MoO_3 (h-MoO_3) phases.

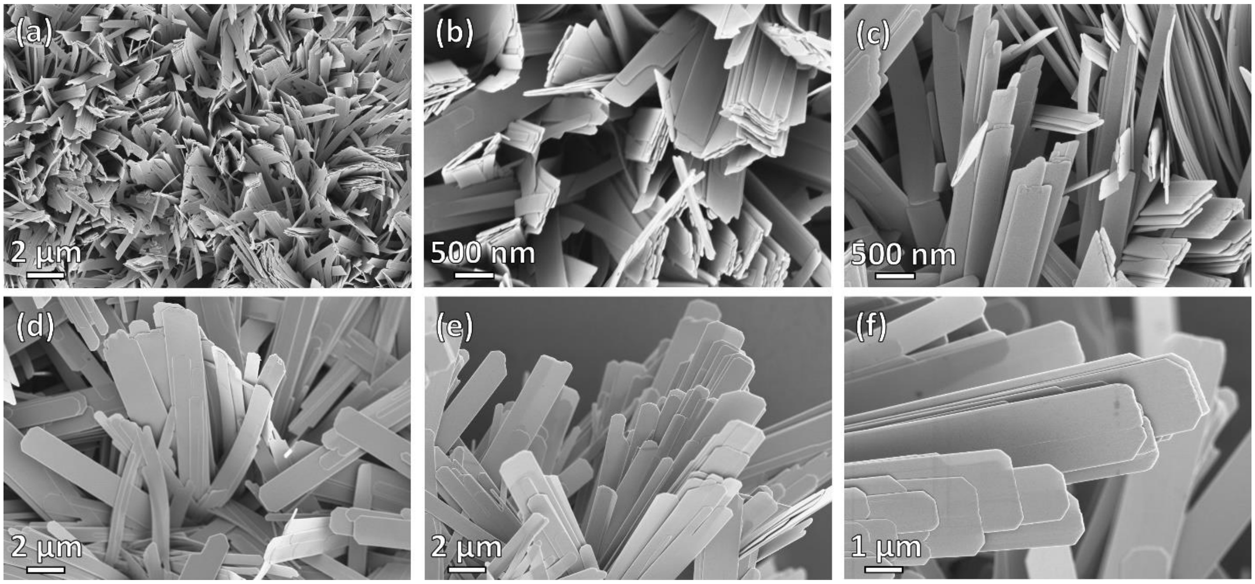

Figure S2. Additional SEM images of the HT-MoO_3/C nanobelts.

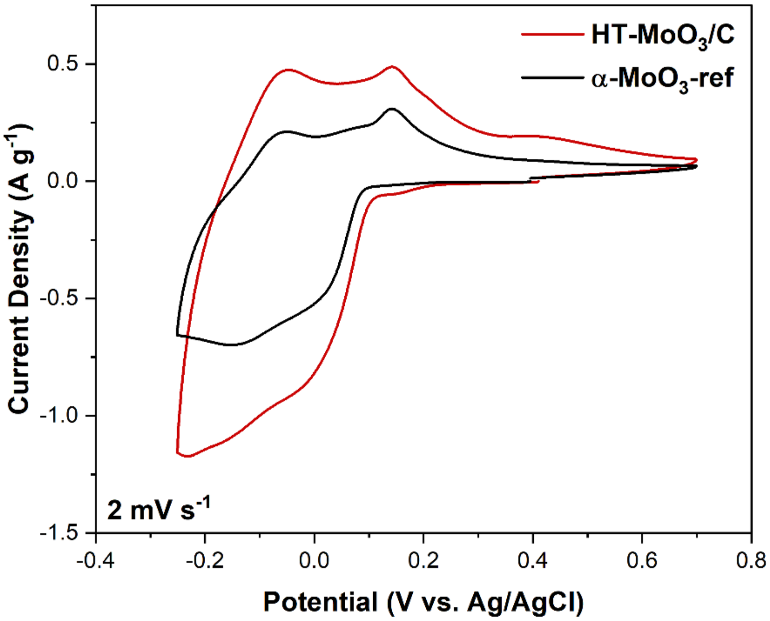

Figure S3. First cycle cyclic voltammograms of HT-MoO_3/C and α-MoO_3-ref electrodes in cells containing 5M $ZnCl_2$ aqueous electrolyte in a potential window from -0.25V to 0.5 V (scan rate of 2 mV s^{-1}).

Four-probe conductivity measurements

Samples preparation for conductivity measurements

To evaluate the electronic conductivity, the resistance of a rolled film was measured using a four-point probe method. Graphene nanoplatelets (GNPs) were added to the sample to enhance conductivity. Samples were prepared by adding 80 wt. % of α-MoO_3-ref or HT-MoO_3/C powders and 15 wt. % of GNPs (xGnP® Graphene Nanoplatelets – Grade M) to the mortar, followed by grinding with a pestle for 10min (**Figure S4a**). The homogenous powder was added to a container, and 5 wt. % PTFE solution in ethanol (**Figure S4b**) were added and mixed in a rotary mixer (FlackTek™) at 3000 rpm (**Figure S4c**). Ethanol was used to uniformly mix the components and to achieve suitable wetting. The mixture was transferred onto a glass slide and rolled using a glass rod to prepare a uniform film with a desired thickness (**Figure S4d** and **S4e**). The thickness of the film was between 0.18 mm and 0.2 mm. The prepared samples were brought into contact with the Jandel instrument head (**Figure S4f**).

The electronic conductivity of the samples was evaluated based on the measured resistance of the rolled films using a Jandel Engineering Ltd four-point probe. The details and a pictorial representation of the steps involved to prepare samples for the sheet resistance measurement is provided in **Figure S4**. A Jandel RM3000+ four-point probe test unit (Jandel Engineering, United Kingdom) with a 1 mm probe distance was used. The film resistivity was measured at currents between 10nA and 1 mA. The reported conductivity was determined via the following equation:

$$\sigma\ (\mathrm{S\ cm^{-1}}) = \frac{1}{4.352\frac{V}{I}t}$$

where V is the measured voltage, I is the applied current, and t is the thickness of the films.

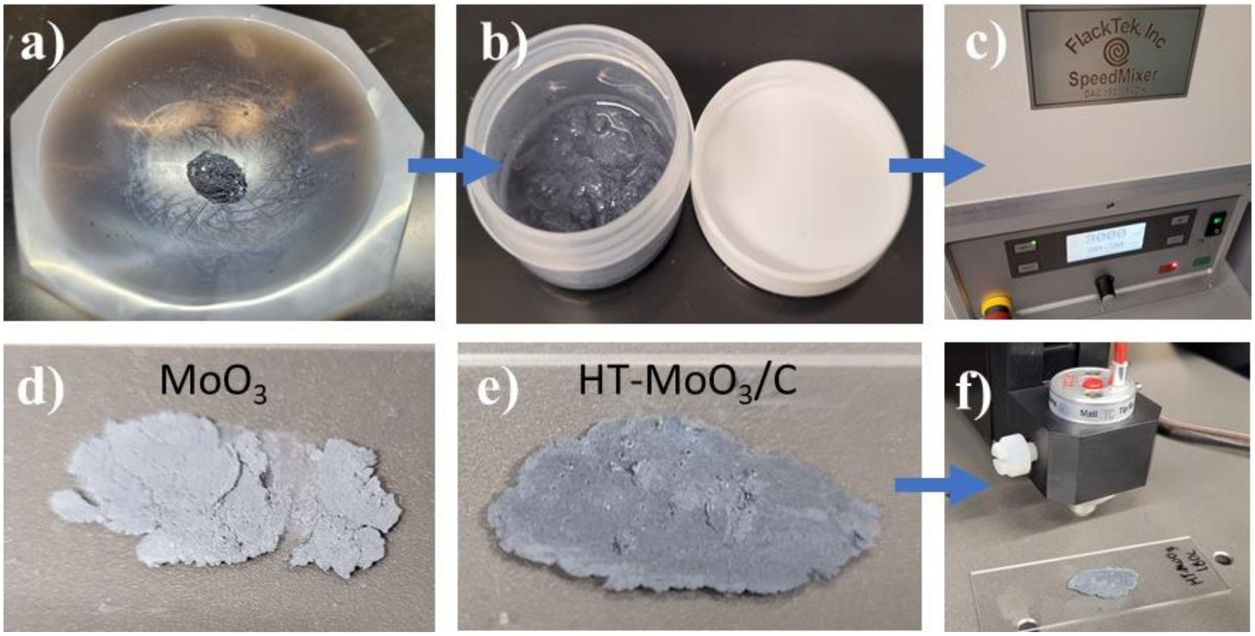

Figure S4. Sample preparation for conductivity measurements: (a) the ground powder, consisting of 80 wt. % of α-MoO_3 or HT-MoO_3/C and 15 wt. of % GNP, (b) the ground powder with 5 wt. % PTFE and ethanol before mixing in a rotary mixer, (c) a rotary mixer (FlackTek™) at 3000 rpm, (d, e) the final (d) α-MoO_3 and (e) HT-MoO_3/C films rolled onto a glass slide, (f) Jandel intrument four-point probe head.

The conductivity measurement results are summarized in **Table S1**. The measurements showed that the conductivity of the HT-MoO_3/C film is five orders of magnitude higher than the conductivity of α-MoO_3-ref. The superior conductivity values of the film can be attributed to the integrated oxide/carbon structure of the HT-MoO_3/C, confirmed via TGA and Raman measurements.

Table S1. Electronic conductivity determined via a four point probe system on the rolled films.

Sample	Electronic Conductivity (S cm^{-1})
α-MoO_3-ref	2.9×10^{-6}
HT-MoO_3/C	5.9×10^{-1}

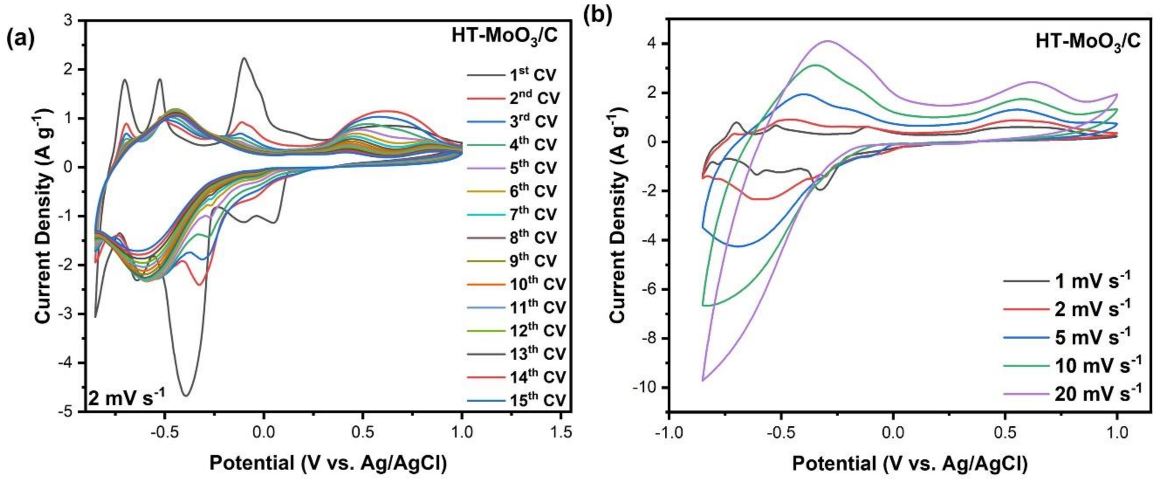

Figure S5. Electrochemical performance of the HT-MoO_3/C electrodes in 5M $ZnCl_2$ aqueous electrolyte in a potential window from -0.85V to 1.00 V. (a) Cyclic voltammetry curves of the cells at a scan rate of 2 mV s^{-1}; and (b) cyclic voltammetry curves at increasing scan rates (1, 2, 5, 10, and 20 mV s^{-1}).
